# Toll-Like Receptor 8 Ligands Activate a Vitamin D Mediated Autophagic Response that Inhibits Human Immunodeficiency Virus Type 1

**DOI:** 10.1371/journal.ppat.1003017

**Published:** 2012-11-15

**Authors:** Grant R. Campbell, Stephen A. Spector

**Affiliations:** 1 Department of Pediatrics, Division of Infectious Diseases, University of California San Diego, La Jolla, California, United States of America; 2 Rady Children's Hospital, San Diego, California, United States of America; National Institutes of Health, National Institute of Allergy and Infectious Diseases, United States of America

## Abstract

Toll-like receptors (TLR) are important in recognizing microbial pathogens and triggering host innate immune responses, including autophagy, and in the mediation of immune activation during human immunodeficiency virus type-1 (HIV) infection. We report here that TLR8 activation in human macrophages induces the expression of the human cathelicidin microbial peptide (CAMP), the vitamin D receptor (VDR) and cytochrome P450, family 27, subfamily B, polypeptide 1 (CYP27B1), which 1*α*-hydroxylates the inactive form of vitamin D, 25-hydroxycholecalciferol, into its biologically active metabolite. Moreover, we demonstrate using RNA interference, chemical inhibitors and vitamin D deficient media that TLR8 agonists inhibit HIV through a vitamin D and CAMP dependent autophagic mechanism. These data support an important role for vitamin D in the control of HIV infection, and provide a biological explanation for the benefits of vitamin D. These findings also provide new insights into potential novel targets to prevent and treat HIV infection.

## Author Summary

Cells use macroautophagy (autophagy - ‘*self-eating*’, lysosome-dependent degradation and recycling of intracellular components in response to stress) as a mechanism to detect intracellular pathogens through pattern-recognition receptors such as Toll-like receptors (TLRs) that recognize signature molecules of pathogens that are essential for their survival. One such Toll-like receptor, TLR8, which is located in human macrophage endosomes, recognizes both imidazoquinoline compounds and uridine-rich single-stranded RNA such as human immunodeficiency virus type-1 (HIV) single-stranded RNA. In the present study we report that TLR8 activation in human macrophages induces the expression of the human cathelicidin microbial peptide (CAMP), the vitamin D receptor (VDR), and cytochrome P450, family 27, subfamily B, polypeptide 1 (CYP27B1), which 1*α*-hydroxylates the inactive form of vitamin D, 25-hydroxycholecalciferol, into its biologically active metabolite. Moreover, we demonstrate that TLR8 activation induces autophagy in human macrophages through a vitamin D and CAMP dependent mechanism, and that the induction of autophagy by TLR8 agonists inhibits HIV. These data support an important role for vitamin D in the control of HIV infection, and provide a biological explanation for the benefits of vitamin D. These findings also provide new insights into potential novel targets to prevent and treat HIV infection.

## Introduction

As an obligatory intracellular parasite, human immunodeficiency virus type-1 (HIV) survival is dependent upon its ability to exploit host cell machinery for replication and dissemination, and to circumvent cellular processes that prevent its growth. One such intracellular process is macroautophagy (hereafter referred to as autophagy). Autophagy is a degradation pathway whereby cytosolic double membrane-bound compartments termed autophagosomes engulf cytoplasmic constituents such as sub-cellular organelles and microbial pathogens. These autophagosomes then fuse with lysosomes, resulting in the degradation of the engulfed components. HIV relies on several components of autophagy for its replication with silencing of autophagy proteins inhibiting HIV replication [1]–[6]. In macrophages, HIV group-specific antigen (Gag)-derived proteins colocalize and interact with microtubule-associated protein 1 light chain 3B (LC3B), and are present at LC3B-II enriched membranes suggesting that autophagy may be involved in Gag processing and the production of nascent virions [5]. This is consistent with the hypothesis that HIV is assembled on endocytic membranes that intersect with recycling endosomes [7], [8]. Despite the requirement for autophagy, HIV actively downregulates autophagy regulatory factors, reducing both basal autophagy and the numbers of autophagosomes per cell [9]–[11]. The HIV negative elongation factor (Nef) protein has been shown to protect HIV from degradation by inhibiting autophagosome maturation [5], and enhances HIV replication through interactions with immunity-associated GTPase family M (IRGM) protein. IRGM interacts with the autophagy-associated proteins autophagy related 5 homologue (ATG5), ATG10, LC3B and SH3-domain growth factor receptor-bound protein 2-like endophilin B1, inducing autophagosome formation [6]. However, inducers of autophagy including amino acid starvation, rapamycin, and 1α,25-dihydroxycholecalciferol (1,25D3), the active form of vitamin D, overcome the imposed phagosome maturation block leading to inhibition of viral replication [2], [3], [11]. Interestingly, the HIV envelope glycoprotein expressed on the surface of infected cells has been reported to induce cell death in uninfected bystander CD4^+^ T cells through autophagy [12], [13].

Recent research has focused on the role of autophagy in the innate and adaptive immune systems. Cells use autophagy as a mechanism to detect intracellular pathogens through pattern-recognition receptors (PRRs) which recognize signature molecules of pathogens termed pathogen-associated molecular patterns (PAMPs) that are essential for their survival. There are several classes of PRRs: Toll like receptors (TLRs), retinoic acid-inducible gene-I-like receptors and nucleotide-binding oligomerization domain-like receptors. These PRRs recognize PAMPs in various cell compartments and trigger the release of inflammatory cytokines and type I interferons for host defense [14], [15]. Human TLR2/1 recognizes *Mycobacterium tuberculosis* lipoproteins, and upon activation induces the expression of cytochrome P450, family 27, subfamily B, polypeptide 1 (CYP27B1) which 1*α*-hydroxylates the inactive form of vitamin D3, 25-hydroxycholecalciferol (25D3), into its biologically active metabolite, the steroid hormone 1,25D3. TLR2/1 agonists also induce the activation and upregulation of the vitamin D (1,25D3) receptor (VDR) leading to the induction of the human cathelicidin microbial peptide (CAMP), autophagic flux and the killing of intracellular *M. tuberculosis* [16], [17]. We have recently demonstrated that 1,25D3 inhibits mycobacterial growth and the replication of HIV through the CAMP-dependent induction of autophagy [3].

TLR8 is phylogenetically and structurally related to TLR7 [18], [19] and is expressed in endosomes of myeloid cells such as monocytes, macrophages and myeloid dendritic cells, and in regulatory T cells [20]–[22]. TLR8 recognizes both uridine-rich single-stranded RNA (ssRNA) and imidazoquinoline compounds [23], [24]. Upon stimulation, TLR8 agonists activate nuclear factor kappa-light-chain-enhancer of activated B cells via the myeloid differentiation primary response gene (88) adaptor protein that leads to the induction of a cascade of antiviral effector functions including the induction of autophagy in murine cells [25] and proinflammatory cytokines in human cells [21]. HIV ssRNA encodes for multiple PAMPs that can be recognized by TLR8 expressed in macrophage endosomes [23], [26] and suppresses HIV replication in acute *ex vivo* human lymphoid tissue of tonsillar origin and renders peripheral blood mononuclear cells (PBMC) barely permissive to HIV infection [20]. Interestingly, HIV downregulates interleukin-1 receptor-associated kinase 4, which is essential for virtually all TLR signaling [27].

Despite the immune defense mechanisms that the host deploys against HIV and improved antiretroviral therapies, the virus persists in long-lived cells including macrophages and dendritic cells. Major questions remain as to the mechanism by which TLR8 agonists inhibit HIV and whether HIV antigens can activate autophagy in human cells through TLR8. In the present study, we demonstrate that TLR8 ligands, in the presence of 25D3, inhibit HIV replication in macrophages through a vitamin D and CAMP-dependent mechanism involving autophagy.

## Results

### TLR8 ligands induce autophagy in human macrophages

**Figure 1:**
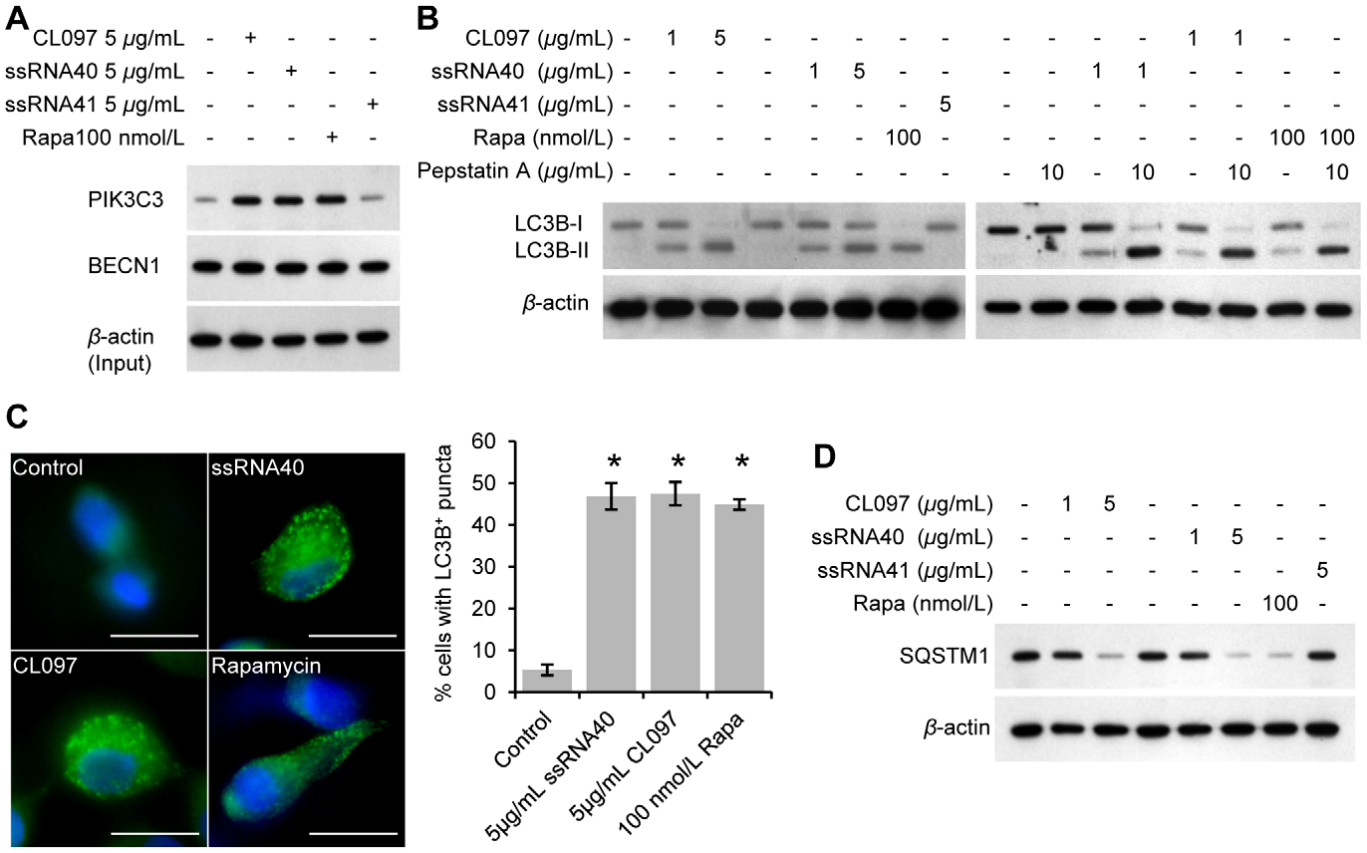
TLR8 ligands induce autophagy in human macrophages. Macrophages were treated for 24 h with CL097, ssRNA40, ssRNA41 or rapamycin (Rapa). (A) Immunoblot of co-immunoprecipitation of cell lysates with BECN1 specific antibody using antibody to PIK3C3 and BECN1 with antibody to *β*-actin as loading control. (B) Cells were incubated with 10 µg/mL pepstatin A for 4 h prior to lysis. Immunoblots of LC3B isoforms using antibody to LC3B or *β*-actin. (C) Cells were fixed and permeabilized then stained with antibody to LC3B (green) and Hoechst 33342 (blue). Scale bars indicate 10 µm. *Left*, representative fluorescence microscopy images. When puncta are not present, LC3B staining gives rise to a diffuse cytoplasmic pattern. *Right*, percentage of cells with LC3B puncta. Bar charts are presented as the means ± s.e.m., *n* = 6. (D) Immunoblots of SQSTM1 using antibody to SQSTM1 or *β*-actin. * *p*<0.05.

Both ssRNA40 and the imiquimod R837 promote autophagic responses in murine RAW 264.7 cells [25] through a beclin-1 (BECN1) dependent mechanism. However, the ability of TLR8 ligands to induce an autophagic response in primary human macrophages has not been investigated. Therefore, the ability of TLR8 agonists to induce autophagy in human macrophages was determined in monocyte-derived macrophages cultured in RPMI 1640 supplemented with 10% (v/v) charcoal/dextran treated, heat-inactivated fetal bovine serum (FBS), 10 ng/mL macrophage colony stimulating factor and 100 nmol/L 25D3 as described in the *Materials and Methods*. The effect of ssRNA40 and the imidazoquinoline CL097 on the formation of the class 3 phosphoinositide-3-kinase (PIK3C3) kinase complex was initially assessed. The PIK3C3 kinase complex is essential for the induction of autophagosome formation at the vesicle elongation step and is formed when BECN1 physically interacts with PIK3C3. Co-immunoprecipitation followed by immunoblotting demonstrated enhanced binding of BECN1 to PIK3C3 forming the PIK3C3 kinase complex following ssRNA40 or CL097 treatment (Figure 1A).

During autophagy, cytosolic LC3B-I is converted to LC3B-II by a ubiquitin-like system that involves ATG7, ATG3 and the ATG12–ATG5 complex. The ATG12–ATG5 complex ligates LC3B-II to the nascent autophagosome membrane through phosphatidylethanolamine with the LC3B-II associated with the inner membrane degraded after fusion of the autophagosome with lysosomes. Therefore, the conversion of LC3B-I to LC3B-II and its turnover is an indicator of autophagy induction and flux [28]. Activation of macrophages with ssRNA40 and CL097 for 24 h led to an increase in LC3B-II similar to that observed with rapamycin, an inducer of autophagy through inhibition of the mammalian target of rapamycin (MTOR) complex 1 (MTORC1), and was increased in the presence of the lysosomal protease inhibitor pepstatin A indicative of autophagic flux (Figure 1B).

During the formation of autophagosomes, LC3B redistributes from a soluble diffuse cytosolic pattern to an insoluble autophagosome-associated vacuolar pattern that can be quantified using fluorescence microscopy [29]. Both ssRNA40 and CL097 induced a significant increase in both the quantity of LC3B per cell and the number of cells with increased LC3B puncta formation in the absence of pyknosis, karyorrhexis, or plasma membrane blebbing and was similar to that observed after rapamycin treatment (Figure 1C). To verify that the increase in the number of autophagosomes in TLR8 agonist treated cells versus control cells represents increased autophagic flux rather than an accumulation of LC3B-positive autophagosomes, the degradation of the polyubiquitin-binding protein sequestosome 1 (SQSTM1) was quantified. Inhibition of autophagy leads to an increase in SQSTM1 protein levels while autolysosomes degrade SQSTM1- and LC3-positive bodies during autophagic flux [30]. Both TLR8 activation and rapamycin treatment of macrophages for 24 h led to a decrease in SQSTM1 protein levels corresponding to the stimulation of autophagic flux (Figure 1D). Moreover, the TLR8 ligands also decreased SQSTM1 protein levels in a dose-dependent manner (Figure 1D).

### Induction of autophagy by ssRNA40 and CL097 is dependent on TLR8

**Figure 2:**
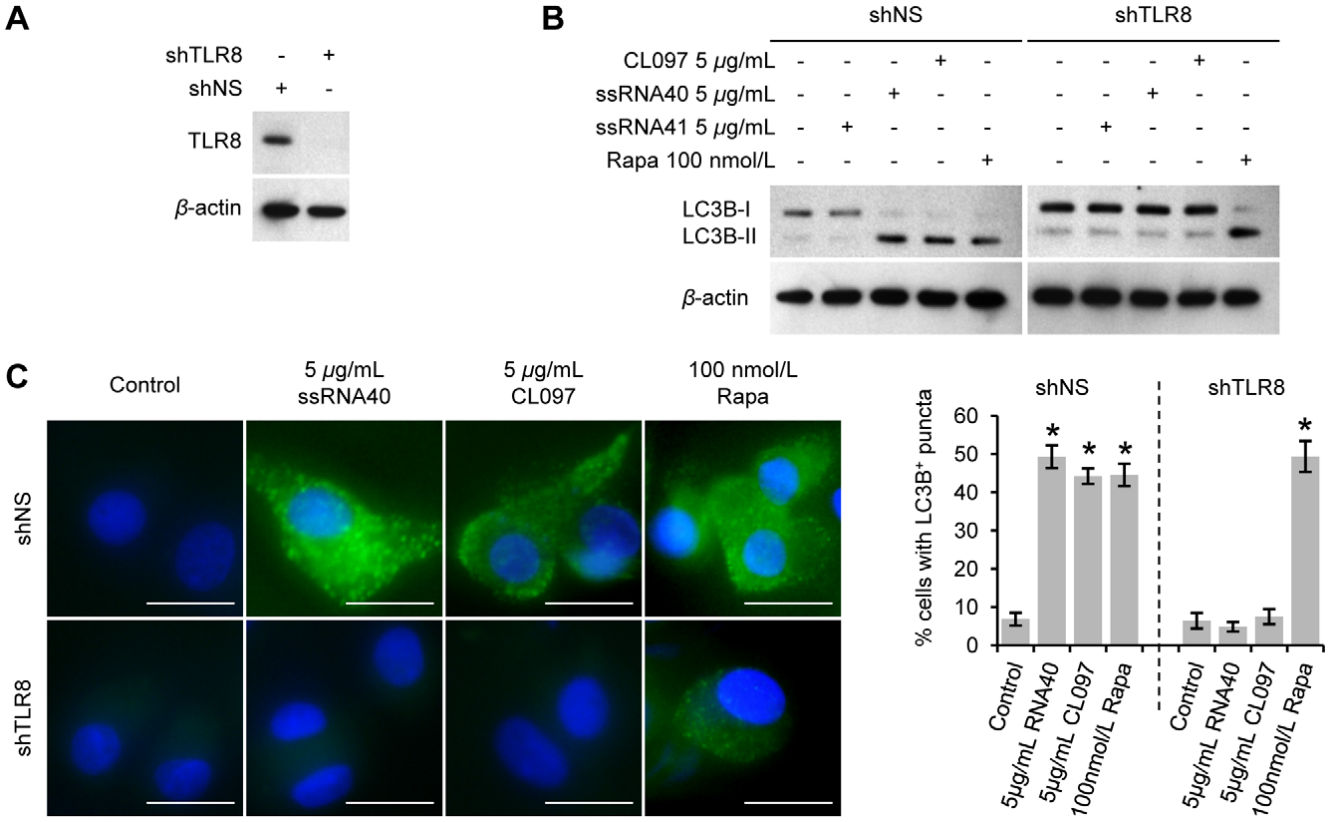
ssRNA40 and CL097 induce autophagy in macrophages through TLR8. Macrophages transduced with non-specific scrambled shRNA (shNS) or TLR8 shRNA (shTLR8) were treated for 24 h with CL097, ssRNA40, ssRNA41 or rapamycin (Rapa). (A) Cells were lysed and immunoblot performed with antibodies to TLR8 and *β*-actin. (B) Immunoblots of LC3B isoforms using antibody to LC3B or *β*-actin. (C) Cells were fixed and permeabilized then stained with antibody to LC3B (green) and Hoechst 33342 (blue). Scale bars indicate 10 µm. *Left*, representative fluorescence microscopy images from three donors are shown. When puncta are not present, LC3B staining gives rise to a diffuse cytoplasmic pattern. *Right*, percentage of cells with LC3B puncta. Bar charts are presented as the means ± s.e.m., *n* = 4. * *p*<0.05.

TLR8 and TLR7 both contribute to the recognition of viral ssRNA and are both found in human macrophages [23], [31]. Therefore, the role of TLR8 and TLR7 in the induction of autophagy in macrophages post-ssRNA40 and CL097 was examined. RNA interference (RNAi) of TLR8 (Figure 2A) significantly inhibited both the ssRNA40- and CL097-mediated LC3B lipidation (Figure 2B) and the increase in the number of LC3B-positive autophagic vesicles (Figure 2C). Although TLR7 is involved in sequence-specific sensing of ssRNAs in human macrophages [31], TLR7 protein expression was undetectable in primary macrophages; therefore, the role of TLR7 in this system is unknown. However, the complete abrogation of TLR8 agonist-induced LC3B lipidation in the presence of RNAi for TLR8 suggests that TLR8 is the mediator of the effect of ssRNA40 and CL097 on autophagy induction.

### TLR8-mediated autophagy requires CEBPB-mediated induction of CYP27B1

**Figure 3:**
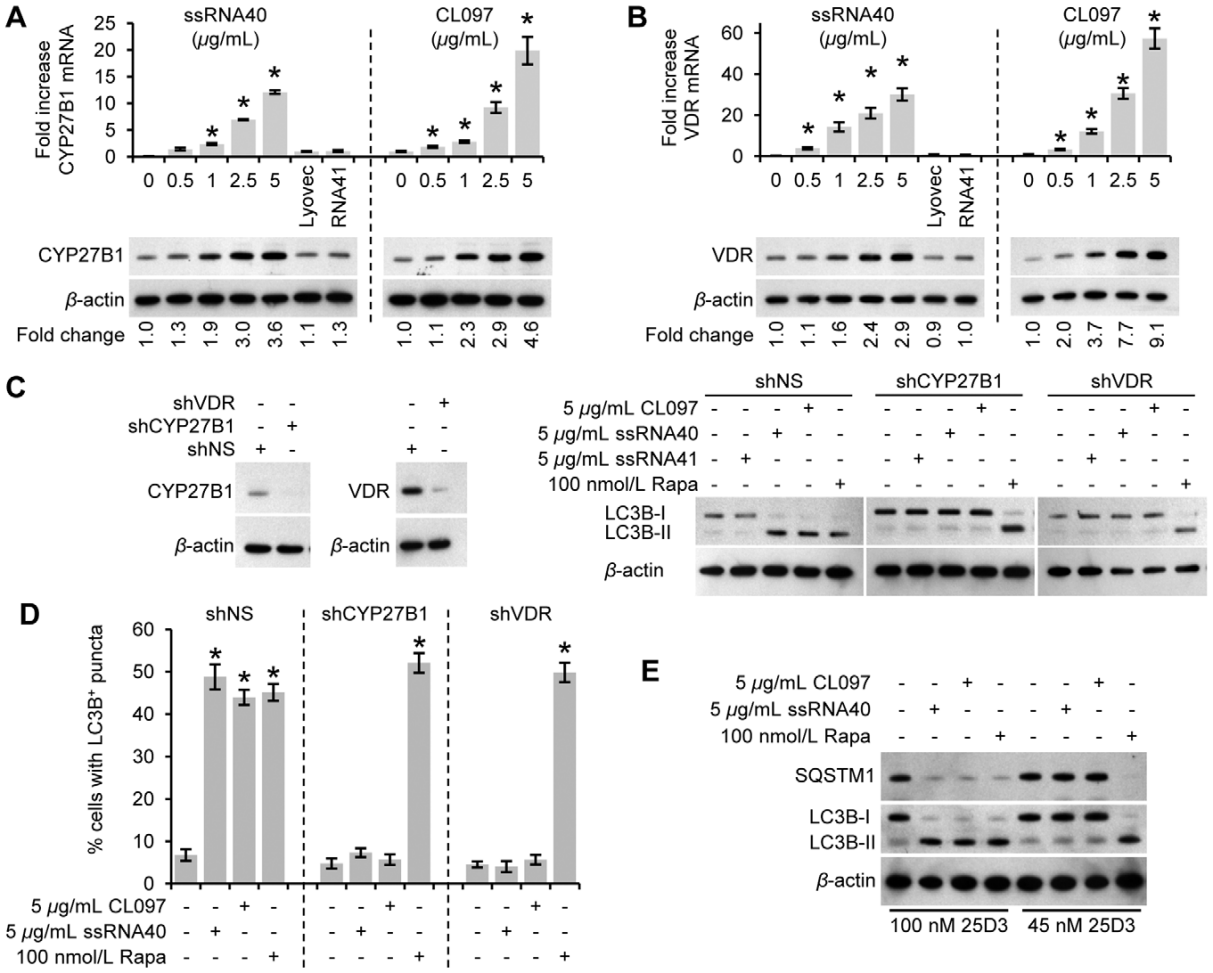
ssRNA40- and CL097-induced autophagy in macrophages is mediated through CYP27B1 and the VDR. (A–B) Macrophages were treated for 24 h with CL097, ssRNA40 or ssRNA41. (A) *Top*, qRT-PCR for CYP27B1 after 6 h. *Bottom*, immunoblots of CYP27B1 using antibody to CYP27B1 or *β*-actin. (B) *Top*, qRT-PCR for VDR after 6 h. *Bottom*, immunoblots of VDR using antibody to VDR or *β*-actin. (C–D) Macrophages transduced with non-specific scrambled shRNA (shNS), CYP27B1 shRNA (shCYP27B1) or VDR shRNA (shVDR) were treated for 24 h with CL097, ssRNA40, ssRNA41 or rapamycin (Rapa). (C) *Left*, immunoblots performed with antibodies to CYP27B1, VDR and *β*-actin. *Right*, immunoblots of LC3B isoforms using antibody to LC3B or *β*-actin. (D) Cells were fixed and permeabilized then stained with antibody to LC3B. Bar charts represent the percentage of cells with LC3B puncta and are presented as the means ± s.e.m., *n* = 4. (E) Macrophages were treated for 24 h with CL097, ssRNA40, and rapamycin in the presence of 45 nmol/L or 100 nmol/L 25D3 and lysed. Immunoblots of LC3B isoforms and SQSTM1 using antibody to LC3B, SQSTM1 or *β*-actin. Bar charts are reported as mean ± s.e.m., *n* = 4. * *p*<0.05.

Recent studies have demonstrated that TLR2/1 activation of human monocytes/macrophages upregulates the expression of vitamin D related genes including CYP27B1 [17] and that 1,25D3 induces autophagy [2], [32]. Given this background, the effect of ssRNA40 and CL097 stimulation on the expression of CYP27B1 and the VDR in macrophages and the role of the vitamin D pathway in TLR8-mediated autophagic flux was investigated. The TLR8 agonists induced a dose-dependent increase in both CYP27B1 (Figure 3A) and VDR (Figure 3B) mRNA and protein expression. Macrophages were then transduced with short-hairpin RNA (shRNA) specific to CYP27B1, VDR or a scrambled non-specific control followed by TLR8 stimulation. Figure 3C shows that CYP27B1 silencing abrogates the lipidation of LC3B in response to TLR8 activation but not in response to rapamycin. Similar results were observed post-VDR silencing. Furthermore, LC3 puncta in CYP27B1 or VDR silenced cells post-TLR8 activation was significantly reduced (Figure 3D). To determine whether differences in vitamin D concentration affect the ability of TLR8 agonists to stimulate autophagy induction, the concentration of 25D3 in the media was reduced to 45 nmol/L reflecting the lower levels observed in vitamin D deficient individuals [33]. At this concentration, the TLR8-mediated induction of autophagy was significantly impaired with little to no LC3B lipidation observed (Figure 3E).

**Figure 4:**
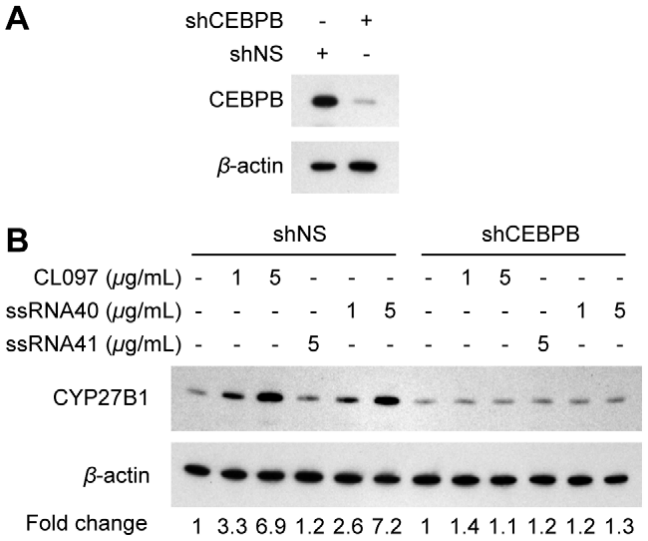
CYP27B1 induction in response to TLR8 ligands is CEBPB-dependent. Macrophages transduced with non-specific scrambled shRNA (shNS) or CEBPB shRNA (shCEBPB) were treated for 24 h with CL097, ssRNA40, or ssRNA41. (A) Cells were lysed and immunoblot performed with antibodies to CEBPB and *β*-actin. (B) Immunoblots of CYP27B1 expression using antibody to CYP27B1 or *β*-actin.

CCAAT/enhancer binding protein *β* (CEBPB) activation is thought to be a required transcription factor controlling immune-mediated transcription of CYP27B1 [34]. Therefore, to assess the role of CEBPB in CYP27B1 expression, macrophages were transduced with shRNA specific to CEBPB, followed by TLR8 stimulation. Figure 4 shows that CEBPB silencing significantly reduced the expression of CYP27B1 in macrophages post-TLR8 activation.

### TLR8 agonists inhibit HIV replication in human macrophages

**Figure 5:**
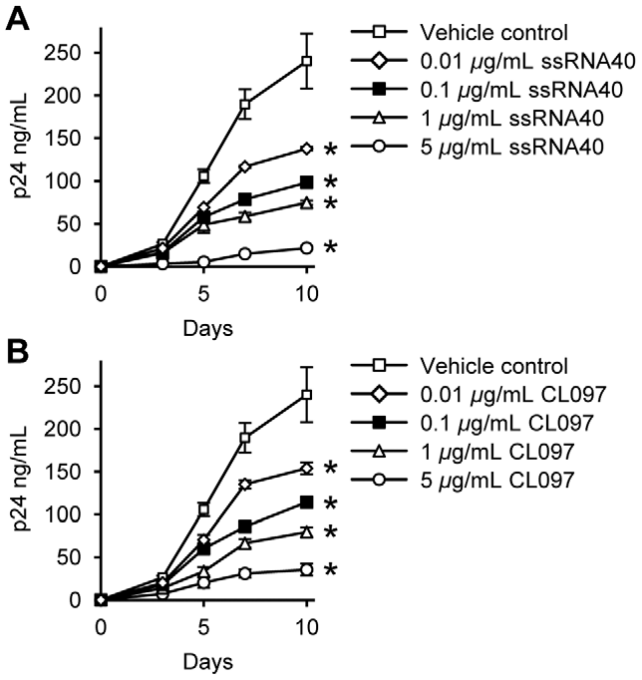
TLR8 agonists inhibit HIV replication. Macrophages were incubated with (A) ssRNA40 or (B) CL097 for 24 h before infection with HIV for 3 h. Cells were then washed and incubated with ssRNA40 or CL097 for 10 d. Extracellular release of HIV p24 antigen into the cell supernatant at days 0, 3, 5, 7, 10 was detected by ELISA. Results are reported as mean ± s.e.m., *n* = 5. * *p*<0.05.

Previous studies have shown that TLR8 agonists inhibit HIV replication in *ex vivo* infected lymphoid tissue while inducing virion release from transformed cell lines [20], [35]. We therefore determined whether the TLR8 agonists influence HIV infection and replication in primary macrophages by comparing the extent to which CL097 and ssRNA40 pre-treatment influenced p24 antigen accumulation in the supernatants of macrophages that were subsequently infected with HIV. Both ssRNA40 and CL097 induced a dose-dependent inhibition of HIV replication. This inhibition became significant across all concentrations tested by day 3 post-infection (*p*<0.01) with the magnitude of the inhibition increasing until cultures were discontinued on day 10 post-infection (Figure 5).

### TLR8-mediated autophagy inhibits HIV replication in human macrophages

**Figure 6:**
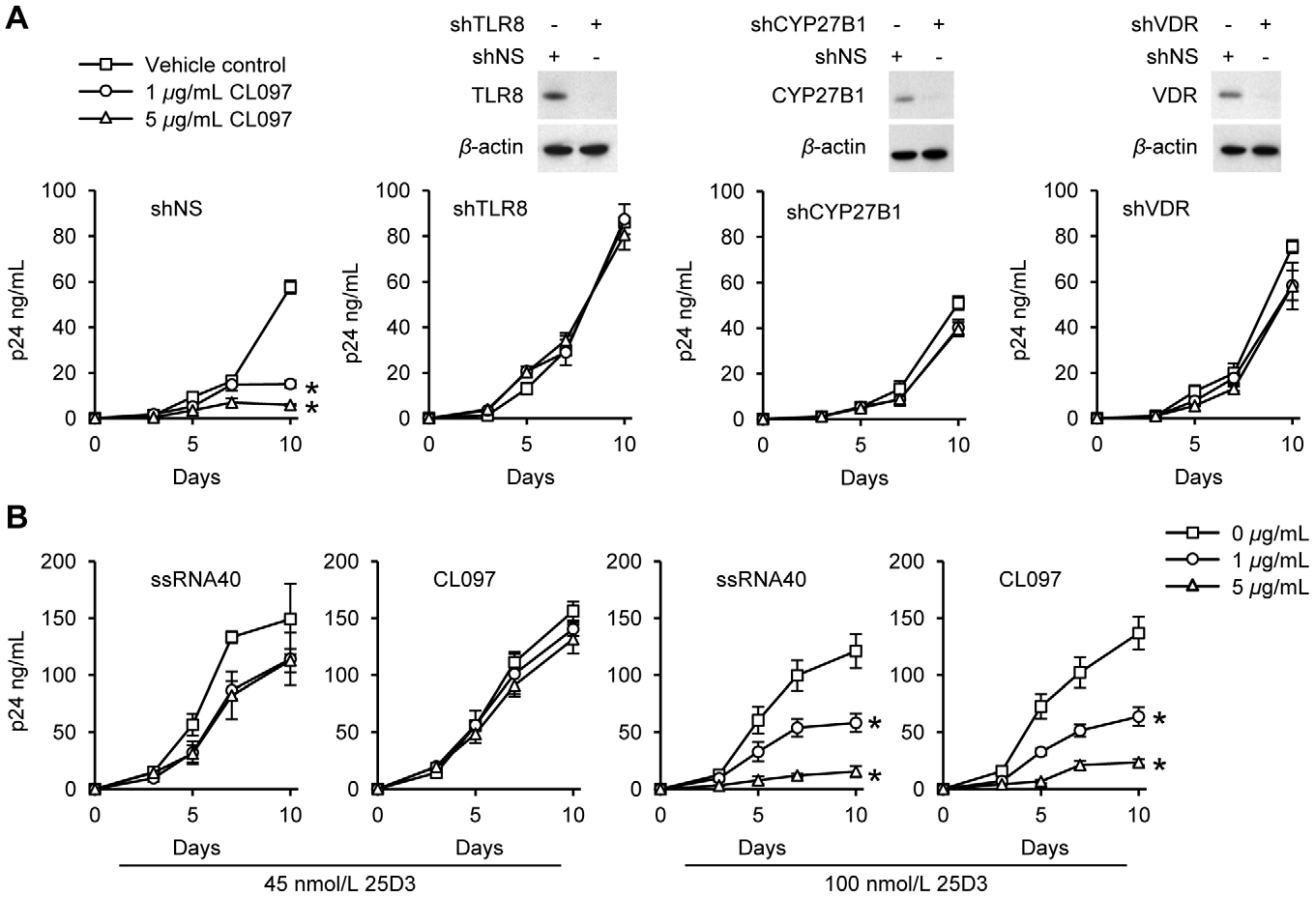
TLR8 agonist inhibition of HIV dependent upon a functional vitamin D pathway. (A) Macrophages transduced with non-specific scrambled shRNA (shNS), TLR8 shRNA (shTLR8), CYP27B1 shRNA (shCYP27B1), or VDR shRNA (shVDR) were treated with CL097 for 24 h before infection with HIV with continuous CL097 treatment. *Top*, immunoblot analysis performed using antibodies raised to TLR8, CYP27B1, VDR and *β*-actin. *Bottom*, ELISA performed for extracellular release of HIV p24 antigen over 10 d. Results are reported as mean ± s.e.m., *n* = 4. (B) Macrophages were incubated with ssRNA40 or CL097 in the presence of 45 or 100 nmol/L 25D3 for 24 h before infection with HIV. Cells were then washed and incubated with 25D3 and ssRNA40 or CL097 for 10 d Extracellular release of HIV p24 antigen was detected by ELISA. Results are reported as mean ± s.e.m., *n* = 6. * *p*<0.05.

To confirm that the inhibition of HIV observed in macrophages post-CL097 stimulation is predominantly through TLR8, we employed RNAi for TLR8. In the scrambled control RNAi treated cells, CL097 inhibited HIV p24 levels by 90% and 74% at 5 and 1 µg/mL, respectively by day 10 post-infection (*p*<0.029; Figure 6A). Conversely, TLR8 silencing reduced the inhibitory effect of CL097 to <6% at both concentrations tested, which was not significantly different to the vehicle control treated cells (*p*>0.48; Figure 6A). Thus, although human macrophages may express low levels of TLR7 [31], TLR8 is the predominant signaling pathway through which CL097 inhibits HIV.

Based on our observations that: i) 1,25D3 inhibits HIV replication through the induction of autophagy [2], [3], ii) TLR8 activation significantly increases the expression of both CYP27B1 and the VDR, and iii) silencing either CYP27B1 or the VDR inhibits TLR8-mediated autophagy, we sought to determine whether the autophagic response induced by TLR8 through the vitamin D pathway was responsible for the observed inhibition of HIV. Silencing of CYP27B1 resulted in the markedly decreased inhibition of HIV by CL097 to levels that were not significantly different to the vehicle control treated cells (*p*>0.05; Figure 6A). Similarly, silencing the VDR significantly reduced the inhibition to control levels (*p*>0.1; Figure 6A) suggesting that the vitamin D pathway is important during the inhibition of HIV by TLR8 agonists. To confirm this, and to determine whether differences in the availability of vitamin D affects the ability of TLR8 agonists to inhibit HIV replication, we reduced the concentration of 25D3 in the media to 45 nmol/L, reflecting the lower levels observed in vitamin D deficient individuals. Under these conditions, we observed a significantly diminished capacity of both ssRNA40 and CL097 to inhibit HIV replication (Figure 6B).

**Figure 7:**
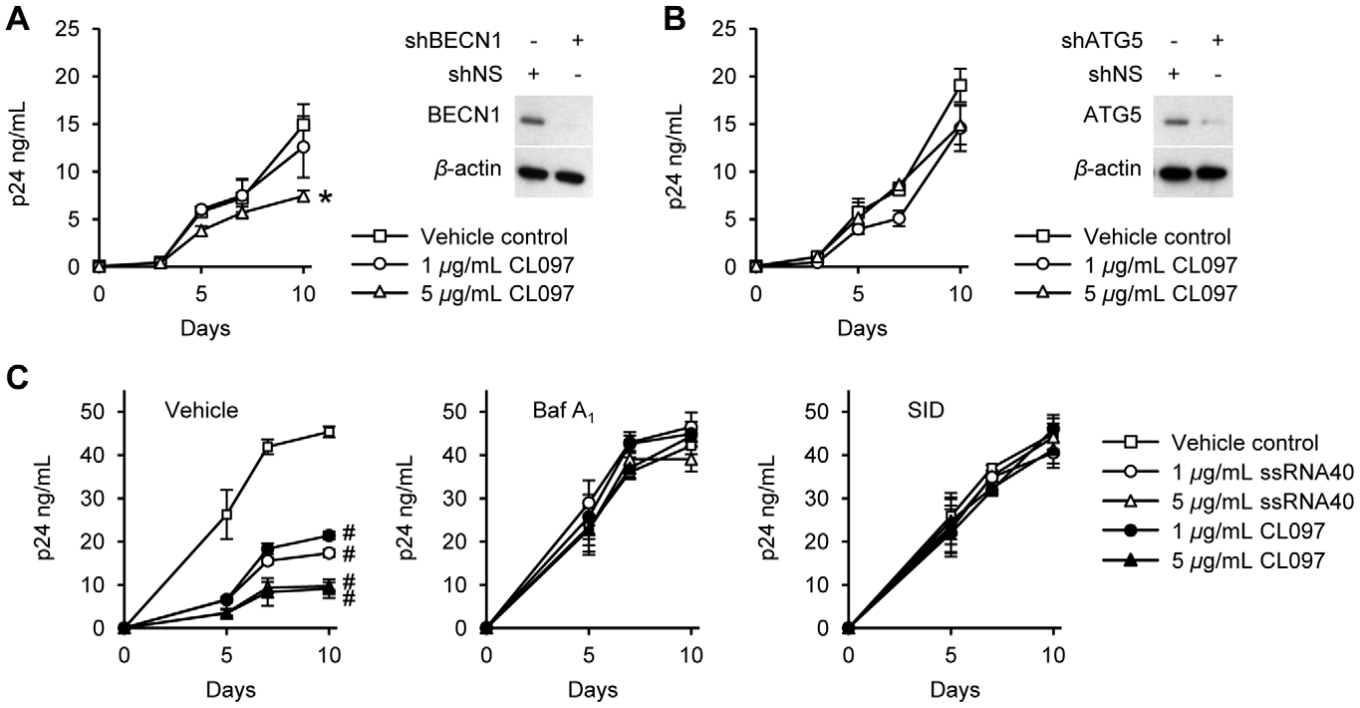
TLR8-mediated inhibition of HIV is autophagy dependent. (A–B) Macrophages transduced with BECN1 shRNA (shBECN1) (A) or ATG5 shRNA (shATG5) (B) were incubated with CL097 for 24 h before infection with HIV with continuous CL097 treatment for 10 d. *Right*, immunoblot analysis performed using antibodies raised to BECN1, ATG5 and *β*-actin. *Left*, ELISA performed for extracellular release of HIV p24 antigen over 10 d. Results are reported as mean ± s.e.m., *n* = 4. (C) Macrophages were pretreated with bafilomycin A_1_ (Baf A_1_), SID 26681509 (SID) or vehicle control before incubation with CL097 or ssRNA40 for 24 h before infection with HIV. Cells were then washed and incubated with TLR8 agonists and inhibitors for 10 d and ELISA performed for extracellular HIV p24 antigen release over 10 d. Results are reported as mean ± s.e.m., *n* = 5. * *p*<0.05; # *p*<0.001.

To determine whether TLR8-induced autophagy contributes to the CL097-mediated inhibition of HIV by CL097, we assessed the effect of BECN1 and ATG5 silencing on HIV infection post-TLR8 activation. BECN1 silencing reduced the 5 µg/mL CL097 mediated inhibition of HIV at day 10 from 90% to 50% (*p* = 0.028; Figure 7A). We next assessed the effect of ATG5 silencing. During autophagy, cytosolic LC3B-I is converted to LC3B-II by an ubiquitin-like system that involves ATG7, ATG3 and the ATG12–ATG5 complex. The ATG12–ATG5 complex ligates LC3B-II to the nascent autophagosome membrane through phosphatidylethanolamine. Therefore, RNAi of ATG5 inhibits autophagosome formation. ATG5 RNAi abrogated the CL097 mediated inhibition of HIV by day 10 (90% *versus* 22% inhibition; *p*<0.028; Figure 7B).

We next investigated whether autophagosome acidification, a late stage event during autophagy, is required for the TLR8-mediated autophagic inhibition of HIV. During autophagy, lysosomes fuse with autophagosomes to form autolysosomes. Macrophages were treated with bafilomycin A_1_, an inhibitor of the vacuolar H^+^ ATPase and autophagosome-lysosome fusion, and subsequently infected with HIV. Bafilomycin A_1_ reversed the TLR8-mediated inhibition of HIV (Figure 7C) suggesting that the acidic pH of autolysosomes is required for the autophagy-mediated control of HIV.

After lysosomes fuse with autophagosomes to form autolysosomes, the sequestered components are degraded by lysosomal hydrolases and released into the cytosol by lysosomal efflux permeases. We investigated whether lysosomal hydrolases are important for TLR8-mediated inhibition of HIV through autophagy using SID 26681509, a novel thiocarbazate specific inhibitor of the lysosome hydrolase cathepsin L. In the absence of TLR8 ligands, SID 26681509 induced no net inhibition of HIV (Figure 7C). Moreover, in the presence of TLR8 ligands, SID 26681509 abrogated the HIV inhibition (Figure 7C).

### TLR8-mediated autophagy and inhibition of HIV is dependent upon the expression of human cathelicidin microbial peptide

**Figure 8:**
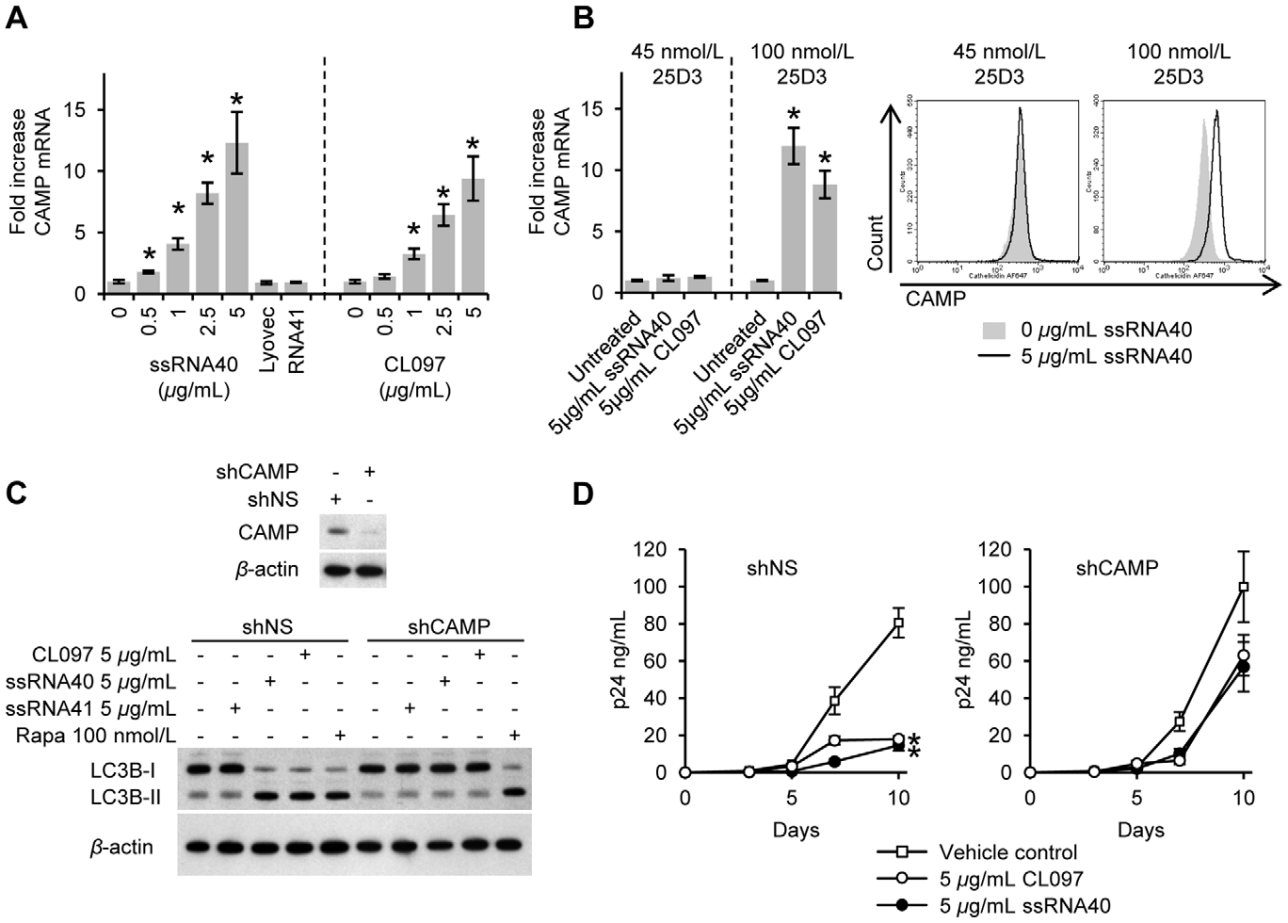
Inhibition of HIV by TLR8 agonists is CAMP and autophagy dependent. (A) Macrophages were treated with CL097, ssRNA40, ssRNA41 for 6 h after which qRT-PCR for CAMP was performed. Results are reported as mean ± s.e.m., *n* = 4. (B) Macrophages were treated for 24 h with CL097 or ssRNA40 in the presence of 45 nmol/L or 100 nmol/L 25D3. *Left*, qRT-PCR for CAMP performed after 6 h. Results are reported as mean ± s.e.m., *n* = 4. *Right*, after 24 h cells were stained with anti-CAMP antibodies and analyzed by flow cytometry. Representative histograms from three donors are shown. (C) Macrophages transduced with non-specific scrambled shRNA (shNS) or CAMP shRNA (shCAMP) were incubated with CL097, rapamycin (Rapa) or vehicle control for 24 h. *Top*, immunoblot analysis performed using antibodies raised to CAMP and *β*-actin. *Bottom*, immunoblots of LC3B isoforms using antibody to LC3B or *β*-actin. (D) After 24 h treatment with CL097 or ssRNA40, macrophages from part C were also washed and infected with HIV and incubated with CL097, ssRNA40 or vehicle control for 10 d. ELISA was performed for extracellular release of HIV p24 antigen over 10 d. Results are reported as mean ± s.e.m., *n* = 6. * *p*<0.05.

Previous studies have demonstrated that CAMP expression is upregulated by 1,25D3, that it is required for 1,25D3 mediated autophagy [3], [32], and that it is involved in the autophagic inhibition of HIV in human macrophages [3]. Moreover, monocytes express CAMP in response to TLR2/1 agonists [16]. Therefore, to determine the role of CAMP in the TLR8-mediated autophagic response, we first investigated whether TLR8 agonists induce the expression of CAMP. Both ssRNA40 and CL097 induced the expression of CAMP mRNA by 13- and 10-fold, respectively over the vehicle control (*p* = 0.029; Figure 8A). These data indicate that TLR8 activation triggers CAMP expression in human macrophages.

A functioning vitamin D signaling pathway is required for the expression of CAMP in response to TLR2/1 agonists [36]. To assess whether TLR8 activation of CAMP expression was dependent on the presence of 25D3, CAMP expression post-TLR8 activation was investigated in macrophages in 25D3 sufficient and deficient media. TLR8-induced CAMP expression was observed in cultures containing 100 nmol/L 25D3, but not in vitamin D deficient culture medium (Figure 8B).

To address the role of CAMP in TLR8-induced autophagy and antimicrobial activity, RNAi for CAMP was employed. Transduction of shCAMP into macrophages significantly blocked endogenous LC3B lipidation post-TLR8 activation, whereas macrophages transduced with a scrambled control (shNS) showed increased LC3B-II conversion consistent with autophagosome formation and the induction of autophagy (Figure 8C). Consistent with the findings that autophagy is required for the restriction of HIV replication, CAMP silencing reduced TLR8-mediated inhibition of HIV to insignificant levels (*p*>0.09; Figure 8D). Collectively, these data suggest that 25D3 is required for the TLR8 induced expression of CAMP and that CAMP expression is required for TLR8-mediated antimicrobial activity in human macrophages.

## Discussion

The antimicrobial effects of vitamin D have been well documented and association studies have linked low levels of 25D3 and/or 1,25D3 with increased risk of, or severity of infection with HIV [37], [38]. The present study identifies how vitamin D deficiency may influence innate immunity against HIV infection. Stimulation of human macrophages with TLR8 agonists upregulates the expression of CYP27B1 and the VDR leading to the induction of CAMP and autophagic flux. Moreover, when serum was 25D3 deficient, or when the vitamin D signaling pathway was silenced, TLR8 agonists were unable to induce autophagy. Thus, the presence of 25D3 and a functional vitamin D signaling pathway are required for TLR8-induced autophagy.

Previous studies have demonstrated that 1,25D3 induces autophagy in primary macrophages through a CAMP dependent mechanism [3], [32] and inhibits HIV replication in macrophages [2], [3]. Consistent with published data, CL097 and the guanosine- and uracil-rich oligonucleotide ssRNA40, but not RNA41 in which all uracils were replaced with adenosines, inhibited HIV replication in primary macrophages [20]. The current study expands on these findings and demonstrates that TLR8 agonists inhibit HIV replication in macrophages through a vitamin D3- and CAMP-dependent mechanism involving autophagy. Indeed, the TLR8-mediated inhibition of HIV replication occurred only in the presence of vitamin D-sufficient media or in cells with an intact vitamin D signaling pathway.

The present data demonstrate that in CAMP silenced cells, TLR8 activation failed to induce LC3B-II lipidation and inhibit HIV. Endogenous CAMP has been implicated in a number of cellular functions including the regulation of inflammatory responses [39] and the formation and maturation of autophagosomes [32]. CAMP has also been shown to play an important role in the activation of mitogen activated protein kinases and CEBPB which contribute to the transcriptional activation of *BECN1* and *ATG5* in response to 1,25D3 [32]. Moreover, during autophagy, autophagosomes recruit CAMP through an AMP kinase, Ca^2+^ and calcium/calmodulin-dependent protein kinase kinase 2 beta dependent mechanism where it is involved in microbial killing [32]. Further work is necessary to determine the precise role of CAMP in TLR-activated autophagy and antiretroviral activity.

Vitamin D deficiency is conservatively defined by most experts as <50 nmol/L 25D3 [33]; 52–72 nmol/L 25D3 is considered to indicate insufficiency and >73 nmol/L considered sufficient [33]. In contrast to this, the estimated mean concentration of 25D3 present in people worldwide is just 54 nmol/L [40]. The major source of vitamin D is through the endogenous photochemical conversion of 7-dehydrocholesterol in the skin to pre-vitamin D3 by ultra-violet B light exposure which then undergoes a 1,7-sigmatropic hydrogen transfer forming cholecalciferol. This is then transferred from the skin by the vitamin D binding protein and is subsequently 25-hydroxylated by cytochrome P450, family 2, subfamily R, polypeptide 1 (CYP2R1) in hepatocytes to form 25D3 in a poorly regulated manner. Lesser amounts of vitamin D3 metabolites are also consumed through fortified dairy products and oily fish. Vitamin D status, therefore, is largely dependent upon the availability of cholecalciferol. Why HIV-infected individuals tend to have lower levels of 1,25D3 and/or 25D3 is largely unknown but it is possible that inadequate renal 1*α*-hydroxylation mediated by pro-inflammatory cytokines and/or a direct effect of antiretroviral drugs play a role [37]. Four genes contribute to the variability of serum 25D3 concentrations: 7-dehydrocholesterol reductase (involved in cholesterol synthesis and the availability of 7-dehydrocholesterol in the skin), 25-hydroxylase CYP2R1, and CYP24A1 (cytochrome P450, family 24, subfamily A, polypeptide 1) (degrades and recycles 1,25D3), and GC (group-specific component [vitamin D binding protein]) which encodes for the vitamin D binding protein. Genetic variations at these loci were recently identified to be significantly associated with an increased risk of 25D3 insufficiency [41].

The characterization of the TLR8/vitamin D mediated antimicrobial mechanism in macrophages provides further evidence of the link between vitamin D and the immune system. In a recent study, 25D3 levels were negatively correlated with the expression of TLR8 in human monocytes. In the same study, it was observed that in healthy individuals circulating 25D3 levels and TLR8 expression decreased with age and that this decrease coincided with a decrease in CAMP expression [42]. Unlike the parathyroid-hormone responsiveness of renal CYP27B1, extra-renal CYP27B1 is not subject to the same feedback control so that the local synthesis of 1,25D3 in macrophages probably reflects the availability of 25D3. Therefore, the intracrine nature of this mechanism suggests that the ability of TLR8 to promote HIV killing could be affected by the availability of 25D3 and the efficiency of the synthesis of 1,25D3 by macrophages.

TLR7 and TLR8 expression in peripheral blood monocytes decreases with disease progression and monocytes from HIV-infected individuals produce less tumor necrosis factor following TLR8 activation than those from uninfected individuals while successfully inhibiting HIV infection [43]. Moreover, these monocyte responses are negatively correlated with CD4^+^ T cell count and positively associated with HIV viral load [44]. The ability of cells to respond strongly to a TLR8 agonist in the presence of high HIV viremia means that ongoing chronic immune activation can be continuously driven by HIV-encoded PAMPs. Despite this, there is no tolerance induction towards TLR8 agonists [35], [44]. Persistent immune activation during HIV infection contributes to the pathogenesis of disease by disturbing the functional organization of the immune system with induction of high levels of cytokines and chemokines. Therefore, chronic stimulation of the innate immune system by TLR ligands may result in the chronic production of proinflammatory cytokines which drive disease progression through generalized immune activation [45]. Supporting this model is the association of a single-nucleotide polymorphism in TLR8 (TLR8 A1G; rs3764880) which confers a significant protective effect against HIV disease progression [46]; however, this same polymorphism increases male susceptibility to pulmonary tuberculosis [47], [48]. Despite these apparent limitations, TLR8 agonists given as vaccine adjuvants with HIV proteins in non-human primate models enhance the magnitude and quality of the anti-HIV Th1 and CD8^+^ T cell responses [49]. Finally, as TLR8 activation of the latently infected cell lines U1 and OM10 results in a marked increase in HIV replication [20], TLR8 triggering of latently infected macrophages may result in the increased release of HIV *in vivo*. Therefore, it may be possible to use TLR8 agonists to purge latently infected cells while inhibiting new infections. Thus, further research on the effect of TLR8 agonists on latently infected macrophages from HIV-infected individuals is warranted.

Collectively, this study demonstrates that TLR8 agonists inhibit HIV replication in macrophages through the induction of autophagy that is dependent upon both available 25D3 and a functioning vitamin D signaling pathway as well as the induction of CAMP. Moreover, this study also expands the known PAMP that induce vitamin D-dependent autophagy to include TLR8. Well-controlled clinical trials are needed to determine if vitamin D supplementation is of value as adjunctive treatment in HIV-infected persons. Dissecting the molecular mechanisms by which HIV utilizes autophagy has the potential to lead to the identification of novel drug candidates to prevent and treat HIV infection and related opportunistic infections including tuberculosis.

## Materials and Methods

### Ethics statement

Venous blood was drawn from HIV seronegative subjects using a protocol that was reviewed and approved by the Human Research Protections Program of the University of California, San Diego (Project 08-1613) in accordance with the requirements of the Code of Federal Regulations on the Protection of Human Subjects (45 CFR 46 and 21 CFR 50 and 56). Written informed consent was obtained from all blood donors prior to their participation.

### Cells and reagents

Peripheral blood mononuclear cells (PBMC) were isolated from whole blood of HIV seronegative donors by density gradient centrifugation over Ficoll-Paque Plus (GE Healthcare). PBMC were then incubated overnight at 37°C, 5% CO_2_ in RPMI 1640 (Gibco) supplemented with 10% (v/v) charcoal/dextran treated, heat-inactivated FBS (Gemini Bio-Products) and 10 ng/mL macrophage colony stimulating factor (R&D Systems), after which non-adherent cells were removed by aspiration. Monocyte derived macrophages were obtained by further incubating the adherent population in RPMI 1640 (Gibco) supplemented with 10% (v/v) heat-inactivated FBS and 10 ng/mL macrophage colony stimulating factor (R&D Systems) for 10 d at 37°C, 5% CO_2_. All experiments were performed in RPMI 1640 supplemented with 10% (v/v) charcoal/dextran treated, heat-inactivated FBS, 10 ng/mL macrophage colony stimulating factor and 100 nmol/L 25D3 (Sigma) unless otherwise stated.

CL097, ssRNA40 and ssRNA41 were obtained from Invivogen and were described previously [23]. Pepstatin A, bafilomycin A_1_, SID 26681509 and rapamycin were purchased from Sigma. Bafilomycin A_1_ was used at 100 nmol/L, SID 26681509 at 50 nmol/L, and pepstatin A at 10 µg/mL with pretreatment for 1 h before addition of TLR8 ligands or rapamycin.

### Virus

HIV_Ba-L_ was obtained through the AIDS Research and Reference Reagent Program, from Dr. Suzanne Gartner and Dr. Robert Gallo [50], [51]. Virus stocks and titers were prepared as previously described using the Alliance HIV p24 antigen ELISA (Perkin Elmer) [52]. Cells were infected with 10^5^ TCID_50_/mL HIV_Ba-L_ per 5×10^5^ cells for 3 h after 24 h pretreatment with TLR8 ligands or rapamycin unless otherwise stated.

### Immunoblotting

LC3B (D11), PIK3C3 (D9A5), SQSTM1 (D5E2), BECN1 (D40C5), and ATG5 antibodies were obtained from Cell Signaling; VDR (N-terminal), TLR8 (4C6), and *β*-actin (AC-74) antibodies were from Sigma; CYP27B1 (H-90) antibody was from Santa Cruz Biotechnology. Cell lysates were prepared using CelLytic M (Sigma) supplemented with protease inhibitors (Thermo Scientific). For co-immunoprecipitation, 50 µg anti-BECN1 was immobilized in a coupling gel then 50 µg of the cell lysates were incubated with the antibody-immobilized coupling gel using the ProFound-Co-Immunoprecipitation kit (Thermo Scientific). For immunoblot analyses, cell lysates were resolved using 2-[bis(2-hydroxyethyl)amino]-2-(hydroxymethyl)propane-1,3-diol buffered 12% polyacrylamide gel (Novex) and transferred to polyvinylidene difluoride membranes (Thermo Scientific), followed by detection with the WesternBreeze chemiluminescence kit (Novex) as described previously [52]. Relative densities of the target bands compared to the reference *β*-actin bands were analyzed using ImageJ (NIH).

### Fluorescence microscopy

Cells were fixed and permeabilized in Dulbecco's phosphate buffered saline supplemented with 4.5% (w/v) paraformaldehyde and 0.1% (v/v) saponin for 30 min, washed, then probed with rabbit anti-LC3B (D11) for 30 mins followed by goat anti-rabbit Alexa Fluor 488 conjugated antibodies (Molecular Probes) for 30 mins and counterstained with Hoechst 33342. Cells and LC3B puncta were imaged and counted using an Olympus IX71 inverted fluorescence microscope as described previously [2].

### shRNA transduction

Lentiviral transduction of macrophages with MISSION lentiviral particles containing shRNAs targeting ATG5 (SHCLNV-NM_004849/TRCN0000150940), BECN1 (SHCLNV-NM_003766/TRCN0000033551), CAMP (SHCLNV- NM_004345/TRCN0000118645), CYP27B1 (SHCLNV-NM_000785/TRCN0000064365), TLR8 (SHCLNV-NM_138636/TRCN0000359246), CEBPB (SHCLNV-NM_005194/TRCN0000007440), VDR (SHCLNV-NM_000376/TRCN0000277001), or scrambled non-target negative control (Scr, SHC002V) was performed according to the manufacturer's protocol (Sigma). Macrophages were transduced with non-specific scrambled shRNA (shNS) or target shRNA and selected using puromycin (Gibco). Five days later, cells were analyzed for target gene silencing and used in experiments.

### Real time-PCR

mRNA quantification was measured by real time PCR using the LightCycler 1.5 Instrument and the FastStart RNA Master SYBR Green I kit (all Roche Applied Science). PCR reactions were carried out in a 20 µL mixture composed of 3.25 mM Mn(CH_3_COO)_2_, 0.5 µM of each primer, 1 µL sample and 1-fold LightCycler RNA Master SYBR Green I. Primers were synthesized by Integrated DNA Technologies and were CYP27B1 sense 5′-GTTTGTGTCCACGCTG-3′, antisense 5′-CCCGCCAATAGCAACT-3′; VDR sense 5′-GTTGCTAAACGAGTCAATCC-3′, antisense 5′-AGTAACGGCACGATCT-3′; CAMP sense 5′-CTCGGATGCTAACCTCT-3′, antisense 5′-CATACACCGCTTCACC-3′; polymerase (RNA) II (DNA directed) polypeptide A (POLR2A) sense 5′-GCACCACGTCCAATGACAT-3′, antisense 5′-GTGCGGCTGCTTCCATAA-3′. Reaction mixtures were initially incubated at 61°C for 20 min to reverse transcribe the RNA. Samples were then heated to 95°C for 30 sec to denature the cDNA followed by 45 cycles consisting of following parameters: CYP27B1 5 s at 95°C, 15 s at 55°C and 16 s at 72°C; VDR 5 s at 95°C, 20 s at 58°C and 25 s at 72°C; CAMP 10 s at 95°C, 10 s at 61°C and 7 s at 72°C each with a single fluorescent reading at the end of each cycle followed by a melting curve analysis. To exclude contamination with DNA, Alu-PCR and minus reverse transcriptase controls were performed. Results were calculated using the Pfaffl method [53] and are expressed as the ratio between the target gene and the reference gene POLR2A and normalized so that CYP27B1, VDR and CAMP mRNA expression in unconditioned cells equals 1.00.

### Flow cytometry

Intracellular staining of endogenous CAMP was performed as previously described [2] using goat anti-LL-37 antibodies (Santa Cruz Biotechnology) and Alexa Fluor 647 conjugated donkey anti-goat antibodies (Invitrogen).

### Statistics

Comparisons between groups were performed using the nonparametric two-sided Mann-Whitney *U* test. Differences were considered to be statistically significant when *p*<0.05.
